# Cultivating cultural awareness among medical educators by integrating cultural anthropology in faculty development: an action research study

**DOI:** 10.1186/s12909-022-03260-7

**Published:** 2022-03-22

**Authors:** Sayaka Oikawa, Junko Iida, Yasunobu Ito, Hiroshi Nishigori

**Affiliations:** 1 Center for Medical Education and Career Development, Fukushima Medical University, 1 Hikarigaoka, Fukushima, 960-1295 Japan; 2 Anthropology, Faculty of Health and Welfare, Kawasaki University of Medical Welfare, 288 Matsushima, Kurashiki, Okayama 701-0193 Japan; 3 Anthropology, School of Knowledge Science, Japan Advanced Institute of Science and Technology (JAIST), 1-1 Asahidai, Nomi, Ishikawa 923-1292 Japan; 4 Center for Medical Education, Graduate School of Medicine, Nagoya University, 65 Tsurumai-cho, Showa-ku, Nagoya 466-8560 Japan

**Keywords:** Faculty development, Cultural anthropology, Cultural awareness, Inquiry-guided reflection, Cultural context

## Abstract

**Background:**

In faculty development, understanding each participant’s cultural context is important. However, there is scarce evidence on how to improve cultural understanding in faculty development. Cultural anthropology is a discipline that focuses on developing cultural self-awareness by understanding different cultures. Professionals from this field can be crucial to the goal of cultivating cultural awareness among medical educators. The aims of this study are to 1) develop and modify cultural anthropology sessions in faculty development and 2) evaluate the effectiveness of these sessions, including their long-term impacts.

**Methods:**

The cultural anthropology sessions were organized as part of a longitudinal faculty development program—Foundation Course for Medical Education—at Kyoto University in Japan. The study included 47 medical educators participating in faculty development and three lecturers: two cultural anthropologists and a medical educator. We developed the cultural anthropology sessions and implemented them in the longitudinal faculty development program. In these sessions, cultural anthropologists used inquiry-guided reflection. An action research methodology was employed and repeated in four cycles from 2015 to 2018. Qualitative and quantitative data were collected during the action research cycles. The qualitative data were thematically analyzed.

**Results:**

The cultural anthropologists’ inquiries fostered learning during the sessions, and three themes—cultural relativism, attention to context, and reframing—were synthesized. As a long-term impact of the sessions, the learners reported becoming more aware of the cultural contexts in their daily educational and clinical activities.

**Conclusions:**

The cultural anthropology sessions in the faculty development program were shown to have enhanced the participants’ awareness of cultural contexts. The concept and format of these sessions may be used more widely in faculty development programs.

**Supplementary Information:**

The online version contains supplementary material available at 10.1186/s12909-022-03260-7.

## Background

It is well known that awareness of cultural context is imperative in medical education, and the importance of considering cultural context during faculty development (FD) has been recognized and discussed more frequently in recent years [1–3]. As FD participants transfer theories, concepts, and models they learned in FD back to their workplaces, FD organizers should consider each participant’s socio-cultural context, for example, where they work, what they teach, and who they are in the medical community. Medical educators who spend most of their time in clinical practices regularly deal with cultural sensitivity and cultural awareness in patient care [4, 5]. However, they are not always accustomed to considering how the cultural context influences their performance when teaching and assessing their students. This is thought to be due to their lack of educational expertise, which can be cultivated by discussing educational issues and reflecting on their teaching [6]. FD programs are the ideal place to help participants gain more awareness on their teaching approaches [7].

Effective FD programs should therefore include a session focused on cultivating participants’ cultural awareness. Cultural anthropology (CA), a discipline that focuses on the study of different cultures, provides a suitable foundation for developing cultural self-awareness in individuals [8] and for improving learners’ objectivity and flexibility when viewing other cultures [9]. Anthropologists strive to understand “cultural others” through ethnographic methodologies [10]. Moreover, in recent years, anthropological insights have begun to be used to solve problems in business, education, and health services [11, 12]. As experts in understanding different cultures, these professionals are crucial to cultivating cultural awareness among medical educators.

The history of medical education recognizes that methods and theories of medical education—many of which were developed in Western culture—have been introduced to other countries. However, such methods and theories have not always been successful in the highly context-dependent field of medical education [13]. Medical educators should be conscious of the uncritical introduction of such methods and theories in medical education. Therefore, we considered that FD programs should provide opportunities to learn about “self-relativization” and turned our attention to CA, which is a discipline of intercultural understanding. Following Stes et al. [14], who suggested the possibility of effective alternative educational formats for FD, the authors developed an innovative inquiry-guided reflection session in collaboration with cultural anthropologists and incorporated it as part of an FD program.

In this study, the authors developed a CA session to cultivate cultural awareness among medical educators and implemented the same in a longitudinal FD program. The aims of this study are to 1) develop and modify the CA session and 2) evaluate the effectiveness of the CA session, including its long-term impact.

## Methods

### Design

**Fig. 1:**
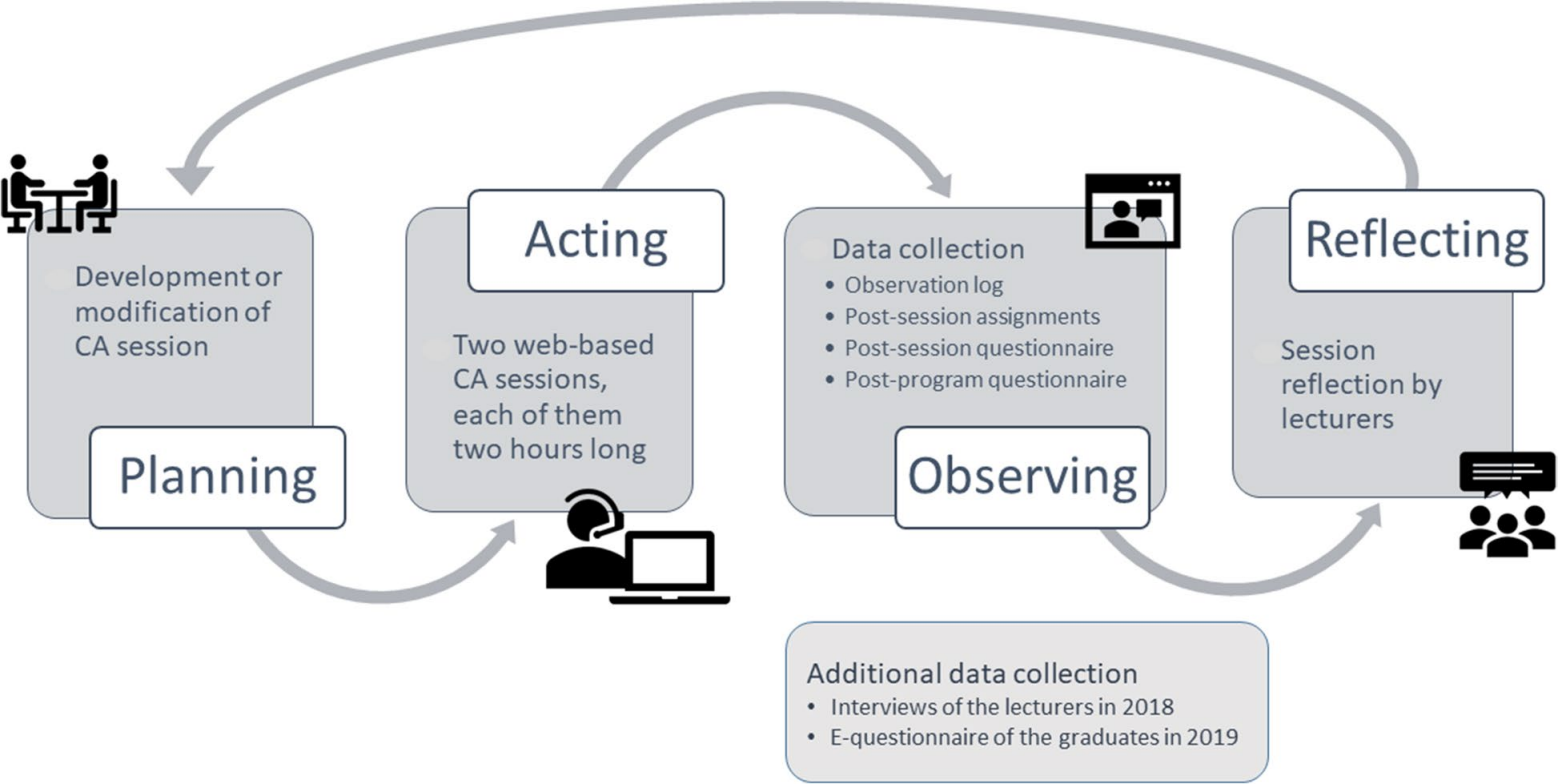
Research flow

We used an action research methodology, which is appropriate for making decisions about program improvement through repetitive reflection, data collection, and analysis [15, 16]. We investigated how the CA sessions were developed and modified, along with the effects of the sessions on participants, by repeating four phases of action research—planning, acting, observing, and reflecting [17, 18]—for four cycles from 2015 to 2018 (Fig. 1).

### Setting and participants

The CA sessions were developed as part of a longitudinal FD program—Foundation Course for Medical Education (FCME)—at Kyoto University in Japan. FCME is a one-year part-time medical education program conducted in web-based and face-to-face sessions. The curriculum contains sessions on teaching and learning, assessments, curriculum development, informatics technology, research, philosophy, leadership and management, general, others, and CA. Twelve participants enroll in the program each year; they are physicians who teach medical students and residents at medical schools or teaching hospitals. The program was first established in April 2015 and over 70 participants have graduated from the program as of 2021.

This study involved 47 participants: 12 each for the classes of 2015, 2016, and 2018, and 11 for the class of 2017. Additionally, the study involved three lecturers: one medical educator and two cultural anthropologists. One participant from the class of 2017 was excluded due to absence from the CA session. The participants were a diverse group of individuals with different ages and areas of specialty (Table 1).

**Table 1:** Demographic data of the participants (Total 47)

Demographic Characteristics	No. (%)
Years of experience	
1–10	14 (29.8)
11–20	23 (48.9)
21–30	9 (19.1)
30-	1 (2.1)
Gender	
Men	35 (74.5)
Women	12 (25.5)
Workplace	
University/University hospital	20 (42.6)
Community hospital	27 (57.4)
Location of workplace	
Japan	
Hokkaido/Tohoku	3 (6.4)
Kanto	16 (34.0)
Chubu	4 (8.5)
Kinki	10 (21.3)
Chugoku/Shikoku	4 (8.5)
Kyushu/Okinawa	9 (19.1)
United States	1 (2.1)
Specialty	
General Internal Medicine	9 (19.1)
Community Medicine/Primary Care	6 (12.8)
Emergency Medicine	6 (12.8)
Pediatrics	5 (10.6)
Cardiology	3 (6.4)
Gastrointestinal Surgery	3 (6.4)
Gastroenterology	2 (4.3)
Psychiatry	2 (4.3)
Cardiovascular Surgery	1 (2.1)
Dermatology	1 (2.1)
Diagnostic Radiology	1 (2.1)
Hematology	1 (2.1)
Intensive Care Medicine	1 (2.1)
Nephrology	1 (2.1)
Neurology	1 (2.1)
Rheumatology	1 (2.1)
Obstetrics and Gynecology	1 (2.1)
Ophthalmology	1 (2.1)
Orthopedic Surgery	1 (2.1)

### Procedures

#### Development and modification of the CA session

In the planning phase of the first year, a medical educator, who is a practicing general internist (HN), and a cultural anthropologist (JI) initiated discussions about drafting CA sessions, establishing learning objectives, scheduling, and educational format. They have collaborated for over a decade in research and teaching settings and obtained successful results [19]. Based on their experiences, they realized that introducing CA into the medical education field will bring the perspective of cultural relativism to medical educators. Therefore, they set the purpose of the sessions as cultivating cultural awareness among medical educators. They created the learning objectives of the sessions as follows: 1) Understanding medical education practice in its social and cultural contexts and 2) developing medical education activities suitable for each context.

As an educational format, they offered a case-based, inquiry-guided reflection. In this format, each participant presented a case of educational practice that they learned in one context and applied in another, with detailed descriptions of their backgrounds; for example, the size, location, or organization of the institutions they learned from and where the practices had been applied. Following the presentations, the participants reflected on how and why their practices succeeded or failed, and the cultural anthropologist lecturer asked questions about the case. By answering the anthropologist’s questions, participants were expected to gain a self-relativization perspective and view themselves differently. In addition to the cultural anthropologist, the medical educator and other participants also asked questions about the case. The CA sessions were web-based, and after the session, each presenter wrote a short reflective essay on the insights gained from the session and submitted it as a post-session assignment. We introduced three books for the participants as references [20–22].

In this case-based, inquiry-guided reflection, participants reflected on the case, and then the cultural anthropologist asked questions to help them reflect further. The developers selected this format because guided reflection is known to help learners develop reflective thinking [23], and the guided interplay between an “internal dialogue” and “external dialogue” is considered necessary for fostering self-reflection [24, 25]. The cultural anthropologists introduced theories and concepts where necessary. Furthermore, we thought that if a cultural anthropologist guides the reflection, their principal methodology of “viewing oneself relatively” would be reflected in the inquiry [11].

Each year, the lecturers reflected on the CA session and, depending on the survey results, modified the contents if necessary.

### Data collection

To assess the lecturers’ modification of the CA sessions, the main author investigated the updated syllabus of the CA session in the planning phases of 2016 to 2018.

To assess the effectiveness of the CA sessions, we collected multiple data in each cycle of action research. In the observation phase of each year, all CA sessions were video recorded; and observation logs, including the qualitative data of participants’ presentation and questions asked by the lectures and other participants, were collected. The post-session assignments, post-session questionnaire, and post-program questionnaire of all participants were also collected via Google forms (see Additional files 1 and 2 for the post-session and post-program questionnaire, respectively).

We also collected data outside the action research cycle. The three lecturers who conducted the CA session in 2018 were interviewed separately, and the interview guide was as follows: What did you keep in mind when teaching the CA session? Why did you keep that in mind?

To assess the long-term impacts of the CA sessions on participants’ educational activities and clinical practices, the author sent an e-questionnaire to the 47 graduates of the FD program in December 2019. The questions for the graduates were: Are you engaged in any new educational activities inspired by CA? Have you made any changes to your practice based on the CA session?

### Data analysis

All qualitative data were transcribed verbatim and thematically analyzed to identify the effectiveness of the CA sessions. We employed an inductive approach to thematic analysis and worked within a constructivist epistemology. Thematic analysis was performed following the six steps of Kiger et al. [26]. The first author (SO), who is a medical educator, documented key words and phrases verbatim and conducted a preliminary thematic analysis, which was reviewed by the co-author (HN). The authors conducted member-checking.

## Results

### Development and modification of the CA session

Several modifications have been made to the CA sessions. In the Class of 2015, there were discussions about different cultural contexts, especially the educational context, such as local educational practices, teaching strategies, and the learners’ attitudes toward education. Thus, the lecturers decided to bring in an anthropologist, specializing in anthropology of knowledge and education, as a lecturer; in 2016, a cultural anthropologist (YI) with experience as an ethnographer in hospital and educational facilities joined the team. Furthermore, the 15 min for presentation and discussion per participant was often insufficient. Therefore, starting from the Class of 2016, the presentation and discussion time per participant was increased to 30 min.

The final model of the CA sessions is shown in Table 2.

**Table 2:** Final model of CA sessions

Title	Cultural anthropology session		
Educational format	Web-based interactive lecture		
Learning objectives	1) To understand medical education practice in its social and cultural contexts 2) To develop medical education activities suitable for each context		
Participants	A cultural anthropologist as a lecturer		
	A medical educator as a mediator		
	Six physicians as learners; three of which are presenters		
Time schedule (Total 2 h)	5 min	Introduction	
	10 min	Presentation of one learner	→ Repeat this cycle for three learners
	20 min	Discussion	
	15 min	Mini lecture on cultural anthropology	
	10 min	Q & A	
Pre-session assignment	The presenters were required to prepare presentation slides describing an instance of taking an educational practice, method, theory, concept, model, or activity that they had learned in one context and applying it in another.		
Post-session assignment	After the session, participants were required to submit a short reflective essay on the session, quoting one or more of the assigned books.		
Assigned books	1)	Jordan, B. and Davis-Floyd, R. (1993) Birth in four cultures: A crosscultural investigation of childbirth in Yucatan, Holland, Sweden, and the United States. 4th Edition. Illinois: Waveland press.	
	2)	Lave, J. and Wenger, E. (1991) Situated Learning: Legitimate Peripheral Participation. Cambridge: Cambridge University Press.	
	3)	Lave, J. (1988) Cognition in Practice: Mind, Mathematics and Culture in Everyday Life. Cambridge: Cambridge University Press.	

### The effectiveness of the CA session

Based on the observation logs, the questions by the anthropologist were divided into two themes: questions about the institutions’ socio-cultural backgrounds and those about their educational practices. The anthropologist asked questions that revealed the cultural contexts in the healthcare setting that medical professionals often take for granted. After elucidating the cultural context of the participants’ cases, the cultural anthropologist asked questions that encouraged the participants’ reflection regarding their educational practices. As an example, the inquiry-guided reflection for Dr. A, an internist, is shown in Table 3.

**Table 3:** An example of the inquiry-guided reflection

Presentation by Dr. A *“At the hospital where I did my residency, I formed a journal club. Now I am working at a community-based hospital that provides acute care. As the members of our department need to treat all patients with gastroenterological problems, we struggle to find the time to either study or engage in academic activities on our own time. For these reasons, we decided to start the journal club to update our medical knowledge. After we started it, however, we encountered several unexpected issues. For example, each participant presented in their way, so the quality of presentations varied. Also, gradually the journal club meetings began to be postponed due to members’ busy schedules and eventually ceased altogether. Based on this experience, I think we should have created general rules for the presentation style. As we were trained in different hospitals during our residency, our experiences at the journal clubs were different. We each tended to conduct the journal club as we did in our residency, and we could not reasonably modify the journal club’s style in our hospital.”* Dialogue between Dr. A and an anthropologist Anthropologist: “*Did you have a similar journal club in your residency*?” Dr. A: “*In the hospital where I trained, the journal club had an established format for presentation. But I was a resident at that time, so I had no experience managing a journal club.”* Anthropologist: *“I need to understand more about the journal club. Where was the venue? How long did the meetings last?”* Dr. A: *“We held the journal club meetings in a conference room for 30 min.”* Anthropologist: *“How did you proceed?”* Dr. A: *“A person in charge read the entire journal and summarized it. They shared this summary and answered questions from other participants.”* Anthropologist: *“I see. You mentioned that each member presented in different ways.”* Dr. A: *“Yes, some of them brought photocopies of the journal while others created presentation slides. Their styles lacked coherence.”* Anthropologist: *“Do you think it matters?”* Dr. A: *“Well, it is a difficult question. I do not think lacking coherence in presentation style really matters. But I think there was inconsistency in the quality of presentations among members, which does matter.”* Anthropologist: *“Hmm. Could you tell me more about the institution you currently work at?”* Dr. A: *“The hospital is located in a prefectural capital and has 400 beds. We accept many emergency cases every day.”* Anthropologist: *“What kind of physicians are in your department?”* Dr. A: *“In our department, I am at the mid-level. There are ten physicians with more experience than me and six physicians who are younger than me.”* Anthropologist: *“How many times did the journal club meet?”* Dr. A: *“About ten times.”* Anthropologist: *“What were the participants’ reactions toward the journal club?”* Dr. A: *“Not so bad, and not so good.”* Anthropologist: *“You mentioned that you could not reasonably modify the journal club in your hospital situation. Please tell me more about this situation.”* Dr. A: *“In our hospital, emergency cases came unexpectedly, so we could not have a regular meeting in the late afternoon…”*	

In terms of how the cultural anthropologist’s inquiries fostered learning during CA sessions, three themes—cultural relativism, attention to context, and reframing—were synthesized. Initials were used to indicate the type of contributor (P = participant and L = lecturer), type of data source (P = post-session assignment, Q = questionnaire, and I = interview), and the year of the class or year of data collection.

#### Theme 1: cultural relativism

Although the participants tried to describe the cultural and contextual differences in detail, it was still accomplished through their lenses as medical professionals. The contextual issues, including differences in the learning environment and learners’ needs or motivation for learning between institutions, were easily shared with peers without detailed explanations, and these were often perceived as a superior-inferior relationship. However, the cultural anthropologist had difficulties understanding such “common perceptions among medical professionals,” and upon inquiry, the presenter noticed differences in their views on culture. Some participants struggled to understand the distinctive aspects of CA, but, gradually, they were able to reflect on the two different contexts as relative rather than as having a superior-inferior relationship. The phrase “cultural relativism” has several variants, including the idea that individuals’ beliefs are relative to the social contexts surrounding them [27], and that individual cultural identity and its effect on relationships are important aspects for physicians [28]. However, in this study, we focus solely on the relative perceptions toward institutions’ socio-cultural contexts, and the culture among medical professions.

A sample response:

When I was teaching learners as an attending physician, I didn’t doubt that the hospital culture I was trained in was superior to the learners’ hospital culture. I think I made many mistakes with that. Even in the same country, there are cultural differences between hospitals. If I had known that there is no superiority or inferiority between cultures, I might have avoided such mistakes. (PP 2017)

#### Theme 2: attention to context

The assigned books and discussion with the cultural anthropologist allowed the participants to understand that different contexts have different practices and that educators should consider the context in which teaching methods are applied. Furthermore, they recognized which teaching method was more appropriate in individual situations and that they should objectively analyze contexts. Throughout this session, it was evident that participants began to focus more on context.

A sample response:

I realized that I focused too much on the methodology of this journal club as a cause of failure. The book ‘Situated learning: Legitimate peripheral participation’ widened my view. Learning should not be an independent activity, and people learn things better within social contexts. This perspective is what I should have had when I introduced a new project. I should also focus on the communities of practice to which the participants belong. For example, learning objectives should be based on the learners’ needs and relevant to their practice situations. I obtained a new viewpoint, “the participants’ situation or context,” by reflecting on my educational activities. (PP 2017)

#### Theme 3: reframing

Based on the reflection, the main role played by the cultural anthropologists in the sessions was to “reframe.” Inquiry-guided reflection by the cultural anthropologist effectively fostered the participants’ self-awareness of their cultural and social contexts by reframing their ways of thinking, beliefs, or positions. Therefore, participants discovered new insights through discussion with the cultural anthropologist.

Sample responses:

The anthropologist’s questions reminded me that what I consider obvious while talking to healthcare professionals may not be obvious for people in other specialty fields. I think the anthropologist lecturers in this session were instrumental in helping me think about understanding others. Because our learners are the others. (PQ 2015).

We can provide the participants with something to shift them from the frame they are using now or give them another viewpoint. Maybe we can do nothing more. I think it would be great if they are encouraged through discussions with anthropologists who specialize in thinking about cultural contexts. (LI 2018).

Interestingly, we [physicians] usually did not ask questions about our educational practice backgrounds, considering the socio-cultural contexts. However, that is important when introducing a new educational activity. The anthropologist asked how many members were in the department, about the venue of the journal club, and details of the hospital’s situation. As physicians, we sometimes ignore these points of view. (LI 2018)

### Long-term impact of CA sessions

Fourteen out of 47 (29.8%) participants responded to an e-questionnaire sent out in 2019; all respondents indicated that the CA sessions impacted their educational activities and practice. In terms of how the CA sessions affected their education and clinical practices, the main theme synthesized in the thematic analysis was intense awareness of context.

Sample responses:

I used to focus on the abilities and qualities of the medical students and residents, but after attending the CA session, I also pay attention to other contexts such as the learners’ background and environment. (PQ 2016)

I became more aware of the cultural context of the patients in the clinical practice. (PQ 2015)

I started a new pre-clinical clerkship session for medical students in collaboration with cultural anthropologists inviting the concept of fieldwork with the learning objective of enhancing cultural context-awareness. (PQ 2016)

**Fig. 2:**
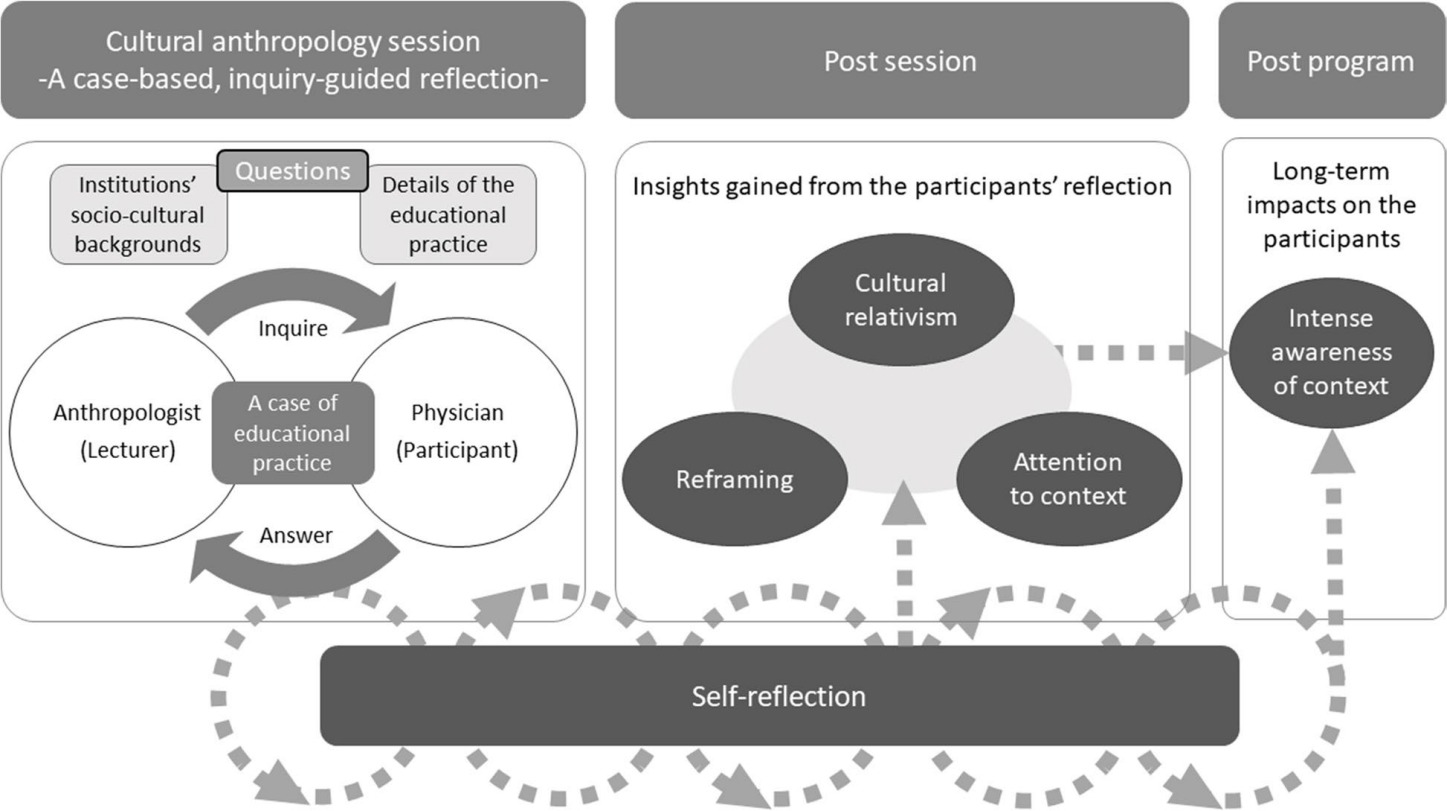
How inquiry-guided reflection led by anthropologists fosters learning and has long-term impacts on participants

Figure 2 illustrates how the inquiry-guided reflection conducted by the anthropologists fosters learning and facilitates long-term impacts.

## Discussion

The authors found that the anthropologist’s questions fostered the learners’ self-reflection by deepening their insight about their educational practices with contextual perspectives. In the CA sessions, unexpected questions about cultural contexts were raised during the inquiry-guided reflection. By answering such questions, the participants’ awareness of “context” developed, and they began to focus on “culture” and “context” more than before. Faculty development activities should employ strategies that stimulate participants’ reflection [7, 29]. However, the facilitators in FD, who play an important role in encouraging learner reflection, are often senior teachers or clinicians [30]. We demonstrated how FD can incorporate participants’ reflections by having experts from non-medical fields collaborate as facilitators.

As the theme of “cultural relativism” suggests, the perspective of the cultural anthropologist had a significant impact on the participants’ realization that there is no superiority-inferiority relationship between the cultures of training hospitals. As to why the participants unconsciously perceived a superiority-inferiority relationship among the training hospitals, the results of previous studies on resident matching programs might have influenced their perceptions. Studies showed that resident applicants placed great importance on the quality of the education provided in the hospital [31], as well as the academic reputation of the program [32]. Based on these results, we might assume that hospitals ranking higher in matching would provide better quality education, and this might affect the physicians’ perspectives regarding the culture of training hospitals. However, a study shows that in recent times, resident applicants are likely to select programs that value residents’ day-to-day interactions and well-being, as opposed to strictly academic factors [33]. Thus, the unconscious perceptions of physicians regarding training hospitals are likely to change in the future, as no association has been found between the ranking of training hospitals and the quality of education provided.

The result of this study showed that being aware of one’s unconscious perceptions of the superiority-inferiority relationship between cultures of training hospitals might be useful, especially when introducing educational practices from one context to another. Cultural relativism could act as a framework based on which educators reflect on their practices when they are unable to conduct these practices as successfully as they expect.

The CA sessions in FD programs enabled an awareness of conditions and circumstances that are often too mundane for the participants to notice. The cultural anthropologist’s purely explorative questions led the participants to review their fixed ideas and norms from a different viewpoint. As previous research has shown, the approach of cultural anthropology has contributed to other specialties by reframing the questions of neuroscience research [34] and mitigating public health misunderstandings [35]. As reflected by the extraction of the theme of “reframing” in our research, cultural anthropology has the potential to add a new perspective to existing fields.

In terms of long-term impact, our study showed that CA sessions might have a certain effect on the participants as educators as well as physicians. The participants increased their awareness of the cultural contexts of not only their students but also their patients. Furthermore, the participants became interested in the discipline of anthropology itself through learning about its theories and concepts. A participant applied the concept of fieldwork to his educational activities by collaborating with the anthropologist. These results have not been described in previous studies about outcomes of FD [7, 36], and we believe that this study added new knowledge to the research on FD.

Considering the above findings, we recommend that FD organizers work with cultural anthropologists to make FD programs more culturally contextual. Collaboration between medical education and cultural anthropology has enormous potential. As the next step, inviting cultural anthropologists into teaching practice settings may be useful, as they could analyze the teaching activities from an ethnographic perspective. Hence, external consultants for FD are recommended for assisting educational change [2]. This strategy will provide medical educators with a deeper insight into their educational activities and can act as an opportunity for them to rethink their education. This approach can be called “workplace-based faculty development,” which has the potential of being more contextual than out-of-workplace FD. Nonetheless, there are several challenges to this approach. First, medical education activities often take place in clinical settings. Therefore, it would be necessary to ensure that cultural anthropologists follow the same patient privacy rules as those followed by healthcare providers. The second challenge would be their limited knowledge of medical and clinical contexts. We, therefore, recommend pairing medical doctors with cultural anthropologists as cultural translators.

### Limitations

This is a single institution study involving 47 graduates. Due to curriculum modification implemented in 2016, the impact of this session on the participants in 2015 and thereon in 2016 may not be the same. In the analysis of long-term impact, we should interpret the results carefully since the response rate was low. In addition, there is a methodological limitation with collecting the graduates’ e-questionnaire once in 2019, which meant that the duration after graduation was not equal for all graduates. Ideally, the graduates’ behavioral changes should be observed in their workplace with an immersion approach after graduation.

## Conclusions

We developed and analyzed a model of FD that includes CA sessions. Our results showed that medical education could learn a lot from CA, as working with individuals from different cultural backgrounds requires self-reflection and personal awareness. Furthermore, our results implied that more collaboration between medical education and CA would enrich our medical education activities, including FD.

## Supplementary Information

**Additional file 1.** Post-session questionnaire.

**Additional file 2.** Post-program questionnaire.

## Data Availability

The data that support the findings of this study are not openly available due to privacy. The materials are available from the corresponding author on reasonable request.

## References

[1] Steinert Y (2011). Commentary: faculty development: the road less traveled. Acad Med.

[2] Steinert Y (2014). Faculty development in the health professions: a focus on research and practice. Vol. 11.

[3] Cilliers FJ, Tekian A (2016). Effective faculty development in an institutional context: designing for transfer. J Grad Med Educ.

[4] Hill RF, Fortenberry JD, Stein HF (1990). Culture in clinical medicine. South Med J.

[5] Bobo L, Womeodu RJ, Knox AL (1991). Principles of intercultural medicine in an internal medicine program. Am J Med Sci.

[6] Seabrook MA (2003). Medical teachers’ concerns about the clinical teaching context. Med Educ.

[7] Steinert Y, Mann K, Anderson B (2016). A systematic review of faculty development initiatives designed to enhance teaching effectiveness: a 10-year update: BEME guide no. 40. Med Teach.

[8] Kleinman A, Benson P (2006). Anthropology in the clinic: the problem of cultural competency and how to fix it. PLoS Med.

[9] Wood DC (2018). EMP at work: disease, health and healing are always cultural: medical anthropology for first-year medical students at Akita University. J Med English Educ.

[10] Flick U (2009). An introduction to qualitative research. 4^th^ ed.

[11] Peoples J, Bailey G (2011). Humanity: an introduction to cultural anthropology. 9^th^ ed.

[12] Ito Y (2019). Contact zone of anthropology of and in business inspiring synergy between anthropology and industry in Japan. Jpn Rev Cult Anthropol.

[13] Lam TP, Lam YY (2009). Medical education reform: the Asian experience. Acad Med.

[14] Stes A, Min-Leliveld M, Gijbels D (2010). The impact of instructional development in higher education: the state-of-the-art of the research. Educ Res Rev.

[15] McMillan JH, Wergin JF (1998). Understanding and evaluating educational research.

[16] Winter R, Munn-Giddings C. A handbook for action research in health and social care: Psychology Press; 2011. p. 3–8.

[17] Cohen L, Manion L, Morrison K (2008). Research methods in education.

[18] Meyer J (2000). Qualitative research in health care. Using qualitative methods in health related action research. BMJ..

[19] Iida J, Nishigori H, Martinez IL, Wiedman D (2021). Managing uncertainly: collaborative clinical case conferences for physicians and anthropologists in Japan. Anthropology in medical education: sustaining engagement and impact.

[20] Jordan B, Davis-Floyd R (1993). Birth in four cultures: a crosscultural investigation of childbirth in Yucatan, Holland, Sweden, and the United States.

[21] Lave J, Wenger E (1991). Situated learning: legitimate peripheral participation.

[22] Lave J (1988). Cognition in practice: mind, mathematics and culture in everyday life.

[23] Stark P, Roberts C, Newble D (2006). Discovering professionalism through guided reflection. Med Teach..

[24] Bindels E, Verberg C, Scherpbier A (2018). Reflection revisited: how physicians conceptualize and experience reflection in professional practice–a qualitative study. BMC Med Educ.

[25] Sandars J (2009). The use of reflection in medical education: AMEE guide no. 44. Med Teach..

[26] Kiger EM, Varpio L (2020). Thematic analysis of qualitative data: AMEE guide no. 131. Med Teach..

[27] Feinberg R (2007). Dialectics of culture: relativism in popular and anthropological discourse. Anthropol Q.

[28] Aggarwal NK, Nicasio AV, DeSilva R, Boiler M, Lewis-Fernández R (2013). Barriers to implementing the DSM-5 cultural formulation interview: a qualitative study. Cult Med Psychiatry.

[29] McLean M, Cilliers F, Van Wyk JM (2008). Faculty development: yesterday, today and tomorrow. Med Teach..

[30] Samarasekera DD, Lee SS, Findyartini A (2020). Faculty development in medical education: an environmental scan in countries within the Asia pacific region. Korean J Med Educ.

[31] Ishida Y, Hosoya Y, Sata N (2012). Educational factors outweigh the importance of lifestyle factors for residency program applicants: an international comparative study. J Surg Educ.

[32] Aagaard EM, Julian K, Dedier J, Soloman I, Tillisch J, Pérez-Stable EJ (2005). Factors affecting medical students' selection of an internal medicine residency program. J Natl Med Assoc.

[33] Yaeger KA, Schupper AJ, Gilligan JT, Germano IM (2021). Making a match: trends in the application, interview, and ranking process for the neurological surgery residency programs. J Neurosurg.

[34] Choudhury S (2010). Culturing the adolescent brain: what can neuroscience learn from anthropology?. Soc Cogn Affect Neurosci.

[35] Marabello S, Parisi ML (2020). "I told you the invisible can kill you": engaging anthropology as a response in the COVID-19 outbreak in Italy. Hum Organ.

[36] Onyura B, Ng SL, Baker LR (2017). A mandala of faculty development: using theory-based evaluation to explore contexts, mechanisms and outcomes. Adv Health Sci Educ.

