# Supplementary material for: Cultivating cultural awareness among medical educators by integrating cultural anthropology in faculty development: an action research study
Source: BMC Med Educ. 2022 Mar 22;22:196. doi: 10.1186/s12909-022-03260-7 (PMC8939140; doi:10.1186/s12909-022-03260-7)
Supplement: Supplementary file 1 — Additional file 1. Post-session questionnaire. [file 12909_2022_3260_MOESM1_ESM.pdf]

Appendix A

Post-session questionnaire

Question 1) Do you want to recommend this session to friends and colleagues who are not participants of the FCME program?
(Strongly agree / Agree / Not sure / Not agree)

Question 2) Please explain why you chose the answer in Question 1.

Question 3) Please write down the positive aspects of this session.

Question 4) Please write down any points about the session that you noticed or that need to be improved

Question 5) Regarding communication during the sessions, Did you have any communication or technical problems?

Question 6) If you noted a problem in Question 5, please describe it. Also, please indicate how the problem was resolved.

Question 7) Please provide feedback on the lecturers. (Please enter any good points, points for improvement, etc.)

Question 8) Please provide any comments regarding the IT team.

Thank you for your cooperation.
