# Supplementary material for: Cultivating cultural awareness among medical educators by integrating cultural anthropology in faculty development: an action research study
Source: BMC Med Educ. 2022 Mar 22;22:196. doi: 10.1186/s12909-022-03260-7 (PMC8939140; doi:10.1186/s12909-022-03260-7)
Supplement: Supplementary file 2 — Additional file 2. Post-program questionnaire. [file 12909_2022_3260_MOESM2_ESM.pdf]

Appendix B

Post-program questionnaire

1) Which one of the sessions did you enjoy most?
   Please tell me why (   )
2) Which one did you learn from and notice most?
   Please tell me why (   )
3) Which were the sessions that most affected your behavior and attitudes?
   Please tell me why (   )
4) Which session would you delete (only one)?
   Please tell me why (   )
5) Which sessions should be dealt with when held for overseas participants?
   Please tell me why (   )
6) Which sessions should be handled in the advanced course?
   Please tell me why (   )
7) If you have any comments about the operation staff and lecturer, please describe.
8) If you have any comments about the environment of the web meeting and equipment, please describe.
9) If you have any comments about the learning support system, please describe.
10) If you have any comments about the duration of the program, please describe.
11) If you have any comments about the size of the participant cohort, please describe.
12) If you have any comments about the assignment, please describe.
13) If you have any comments about the time schedule of the web meeting, please describe.
14) If you have any comments about the extra sessions, please describe.
15) If you have any comments about the accessibility of the library system, please describe.
16) If you have any comments about the open campus, please describe.

Thank you for your cooperation.
